# Comparative analysis of fungal protein kinases and associated domains

**DOI:** 10.1186/1471-2164-11-133

**Published:** 2010-02-24

**Authors:** Idit Kosti, Yael Mandel-Gutfreund, Fabian Glaser, Benjamin A Horwitz

**Affiliations:** 1 Department of Biology, Technion - Israel Institute of Technology, Haifa 32000, Israel; 2 Bioinformatics Knowledge Unit, The Lorry I. Lokey Interdisciplinary Center for Life Sciences and Engineering, Technion - Israel Institute of Technology, Haifa 32000, Israel

## Abstract

**Background:**

Protein phosphorylation is responsible for a large portion of the regulatory functions of eukaryotic cells. Although the list of sequenced genomes of filamentous fungi has grown rapidly, the kinomes of recently sequenced species have not yet been studied in detail. The objective of this study is to apply a comparative analysis of the kinase distribution in different fungal phyla, and to explore its relevance to understanding the evolution of fungi and their taxonomic classification. We have analyzed in detail 12 subgroups of kinases and their distribution over 30 species, as well as their potential use as a classifier for members of the fungal kingdom.

**Results:**

Our findings show that despite the similarity of the kinase distribution in all fungi, their domain distributions and kinome density can potentially be used to classify them and give insight into their evolutionary origin. In general, we found that the overall representation of kinase groups is similar across fungal genomes, the only exception being a large number of tyrosine kinase-like (TKL) kinases predicted in *Laccaria bicolor*. This unexpected finding underscores the need to continue to sequence fungal genomes, since many species or lineage-specific properties may remain to be discovered. Furthermore, we found that the domain organization significantly varies between the fungal species. Our results suggest that protein kinases and their functional domains strongly reflect fungal taxonomy.

**Conclusions:**

Comparison of the predicted kinomes of sequenced fungi suggests essential signaling functions common to all species, but also specific adaptations of the signal transduction networks to particular species.

## Background

Filamentous fungi detect and respond to a variety of signals. As saprophytes or parasites, fungi need to monitor the nutrient status, presence of a host and host defenses, and avoid or respond to osmotic or oxidative stress, light and other environmental variables [1]. Although fungal development is less complex than that of higher multicellular eukaryotes, internal signals are probably required to program major differentiation steps: hyphal extension and branching, sporulation, mating, secondary metabolite accumulation and production of infection structures. Finally, regulation of transcription, translation and cell division is expected to be similar to that of other eukaryotes. Indeed, fungal models such as budding and fission yeasts, *Neurospora *and *Aspergillus *have figured prominently in cell biology and genetics [2].

Protein kinases have roles in every aspect of regulation and signal transduction, and provide new targets for drug development [3]. Most eukaryotic protein kinases, with the exception of the histidine kinases found in two-component sensory systems, belong to a superfamily defined by a conserved protein kinase domain (ePK, eukaryotic protein kinase). There are nearly 500 members of this superfamily in human and mouse [4-6]. The ePK superfamily can be classified into several major groups. The members of each of these groups are related according to the signals that activate them and the kinds of protein substrates they phosphorylate. The original classification of Hanks and Hunter [7] has been extended, refined, and applied to many eukaryotic genomes including some fungi [8]. Beginning a few years ago there has been an increasing effort to sequence filamentous fungal genomes [9]. There has, however, been no complete study of the kinomes of the recently sequenced filamentous fungi. Furthermore, in addition to identifying and classifying the kinases in the genomes, there is now a unique opportunity to discover species-specific properties, as well as general trends related to taxonomic group or other properties that are shared between different sequenced fungi.

Based on the kinomer database [8], we developed an automatic pipeline that predicts all putative kinases from any given proteome, and classifies them. We then analyzed their distribution, and tested different potential classification methods. In addition to the basic ePK domain present in almost all putative kinases, other domains of the protein are essential for kinase activity and interaction with effector proteins and substrates. In the human kinome, 83 additional domain types were identified, and additional domains were recognized in over half of the kinases [6]. We identified all additional domains that are present in the fungal kinomes according to PFAM classification, and discuss their presence or absence in the different groups. Finally, we show that the information contained in the domains is sufficient to classify the fungi. Our analysis can be applied to any other taxonomic or protein groups, and suggests a great functional richness of kinases in different organisms.

## Results and Discussion

We have studied the distribution, domain content and kinase density among 30 species representing the Dikarya or higher fungi, including two phyla (see Table 1). In this analysis, we obtained the full proteome for each fungal genome from diverse sources, and then used the Kinomer database [10] to identify and classify each predicted kinase. This database classifies the eukaryotic protein kinases into two groups: 'conventional' (ePK) and 'atypical' (aPKs) protein kinases. This classification, based on sequence similarity, also allows the construction of an accurate multi-level HMM library that can be used to search and classify each putative kinase in any organism to each of the 12 basic sub-groups (see Methods for details).

**Table 1:** Species included in the kinome analyses.

	Fungal species	Abbreviation	Phylum/Subphylum/Class*	Database	Genome Paper
**1**	*Ascosphaera apis*	Aapis_as	Ascomycota/P/E	Baylor/NCBI	[31]

**2**	*Aspergillus clavatus*	Aclavatus_as	Ascomycota/P/E	NCBI Protein	[32]

**3**	*Aspergillus fumigatus*	Afumigatus_as	Ascomycota/P/E	Broad Institute	[28,33]

**4**	*Aspergillus nidulans*	Anidulans_as	Ascomycota/P/E	Broad Institute	[28,33]

**5**	*Aspergillus niger*	Aniger_as	Ascomycota/P/E	NCBI Protein	[34]

**6**	*Aspergillus oryzae*	Aoryzae_as	Ascomycota/P/E	Broad Institute	[28,33]

**7**	*Coccidioides immitis*	Cimmitis_as	Ascomycota/P/E	Broad Institute	[35]

**8**	*Fusarium graminearum*	Fgraminearum_as	Ascomycota/P/So	Broad Institute	[36]

**9**	*Histoplasma capsulatum*.	Hcapsulatum_as	Ascomycota/P/E	Broad Institute	[35]

**10**	*Magnaporthe oryzae (grisea)*	Mgrisea_as	Ascomycota/P/So	Broad Institute	[37]

**11**	*Neurospora crassa*	Ncrassa_as	Ascomycota/P/So	Broad Institute	[38]

**12**	*Neosartorya fischeri*	Nfischeri_as	Ascomycota/P/E	Venter	[32]

**13**	*Penicillium chrysogenum*	Pchrysogenum_as	Ascomycota/P/E	NCBI Protein	[39]

**14**	*Stagonospora nodorum*	Snodorum_as	Ascomycota/P/D	JGI	[40]

**15**	*Sclerotinia sclerotiorum*	Ssclerotiorum_as	Ascomycota/P/L	Broad Institute	In preparation

**16**	*Trichoderma reesei*	Treesei_as	Ascomycota/P/So	JGI	[41]

**17**	*Uncinocarpus reesii*	Ureesii_as	Ascomycota/P/E	Broad Institute	[35]

**18**	*Cryptococcus neoformans*	Cneoformans_ba	Basidiomycota/A	Broad Institute	[42]

**19**	*Laccaria bicolor*	Lbicolor_ba	Basidiomycota/A	JGI	[43]

**20**	*Malassezia globosa*	Mglobosa_ba	Basidiomycota/U	NCBI Protein	[44]

**21**	*Phanerochaete chrysosporium*	Pchrysosporium_ba	Basidiomycota/A	JGI	[45]

**22**	*Ustilago maydis*	Umaydis_ba	Basidiomycota/U	Broad Institute	[46]

**23**	*Ashbya (Eremothecium) gossypii*	Agossypii_he	Ascomycota/S	NCBI Protein	[47]

**24**	*Candida albicans*	Calbicans_he	Ascomycota/S	Broad Institute	[48]

**25**	*Candida glabrata*	Cglabrata_he	Ascomycota/S	Genolevures	[49]

**26**	*Debaromyces hansenii*	Dhansenii_he	Ascomycota/S	Genolevures	[49]

**27**	*Kluyveromyces lactis*	Klactis_he	Ascomycota/S	Genolevures	[49]

**28**	*Pichia stipitis*	Pstipitis_he	Ascomycota/S	JGI	[50]

**29**	*Saccharomyces cerevisiae*	Scereviseae_he	Ascomycota/S	SGD	[51]

**30**	*Schizosaccharomyces pombe*	Spombe_as	Ascomycota/T	Sanger	[2]

Taxonomy follows [18] and [21]. Saccharomycotina (Ascomycete yeasts) are often referred to as Hemiascomycota and we have retained the tag _he in the abbreviated species names in the figures, for simplicity.

*Subphylum: U, Ustilaginomycotina; A, Agaricomycotina; P, Pezizomycotina; S, Saccharomycotina; T, Taphrinomycotina; Class: So, Sordariomycetes; L, Leotiomycetes, E, Eurotiomycetes; D, Dothideomycetes.

### Distribution of kinases in each functional sub-group

**Figure 1:**
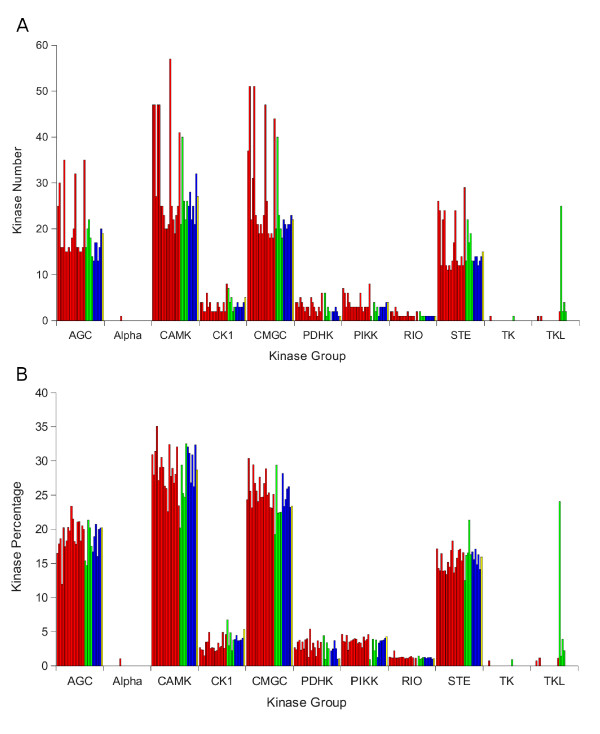
**Classification of predicted protein kinase genes by fungal species**. A) Number of kinases identified in fungal proteomes for each kinase group: AGC, Alpha, CAMK, CK1, CMGC, PDHK, PIKK, RIO, STE, TK, TKL (see [7,8,10]). Species are sorted according to the order of their listing in Table 1. Colors indicate the major phyla/subphyla: red is for Ascomycota, green is for Basidiomycota, blue is for subphylum Saccharomycotina of the Ascomycota and yellow is for *S. pombe *(Ascomycota, subphylum Taphrinomycotina). B) Same as in (A) but for normalized number of kinases (% kinases within the total kinome).

The initial result of this work is a list of putative kinase proteins, classified by the Kinomer library. Figure 1A shows the distribution of the 11 populated groups of kinases (RGC has no representatives) found by applying the Kinomer HMM library to each of the 30 fungal proteomes studied. The main populated groups of kinases are AGC, CMAK, CMGC and STE, all of them belonging to the protein kinase superfamily [7,10]. These four groups include 88% of all predicted kinases on average. The least populated groups are Alpha and TK with only 1 and 2 representatives for all fungi, respectively. Within the Hemiascomycota group (ascomycete yeasts: subphylum Saccharomycotina) the number and distribution of the different kinase groups are generally similar (Figure 1A). In contrast, within the filamentous Ascomycota (subphylum Pezizomycotina) and the Basidiomycota groups, the variation in terms of kinase number is much higher. When we look, however, at the normalized frequency of kinases in each group (relative to the total number of kinases in each fungal proteome, Figure 1B), it is evident that, despite minor variations within the different phyla and subphyla, the overall proportion of each group is quite similar in each kinase sub-group: AGC includes about 20% of all kinases in each species, CAMK about 30%, etc.

Thus, most variation between species diminishes upon normalization. Nevertheless, in terms of the kinome size, we can see two distinct groups. The first one has larger kinomes with, on average, 159 kinases, and includes the ascomycetes *Aspergillus clavatus*, *Aspergillus niger*, *Neosartorya fischeri*, *Aspergillus nidulans *and *Ascosphaera apis *and the basidiomycetes *Cryptococcus neoformans *and *Malassezia globosa*. The second group includes all other fungi in our study, having an average of 85 putative kinases (see Additional File 1). Thus, the larger group has, on average, almost twice the number of kinases compared to the smaller group. The large variation in the total number of kinases might be related to partial genome duplication event(s). This assumption is supported by considerable evidence of genome duplication in fungi [11]. However, expansion of the number of predicted protein kinases does not correspond to the overall predicted frequency of duplications. *Aspergillus nidulans*, for example, has an expanded number of kinases compared to *A. fumigatus *and *A. oryzae *(as we find also in this study, see Additional File 1), yet the estimated frequency of duplications [11] is quite similar for these three members of the genus *Aspergillus*. About a third of our samples seem to have undergone a possible duplication event of either the kinome and/or the genome of these fungi.

As mentioned above, the Hemiascomycota (Ascomycota, Subphylum Saccharomycotina) group seems to be much more compact in terms of kinase number. This indicates, perhaps, a "tighter" evolution of this group with respect to their signaling pathways. For those fungi that have not undergone kinome duplication, the number of kinases found for each group is remarkably similar (see black bars in Additional File 1), given the large evolutionary differences between them. The reasons for independent expansion of protein kinase gene families are not clear, but it has been proposed that genes involved in regulatory interactions might retain duplication. The result would be selective expansion of these classes of genes. Modeling of the effects of three genome duplications in *Arabidopsis *over the past 350 million years suggests that duplications of regulatory genes are retained, particularly, for large-scale events, because signal transducer proteins act in complexes [12,13]. The Zygomycete *Rhizopus oryzae *genome has undergone a high level of duplication [14]. In this species, we found (data not shown) that the main kinase families are expanded about two fold. Here again, after normalization the relative number of kinase families are within the range of the Dikarya which are shown in Figure 1B. Analysis of other Zygomycete species will answer the question of whether expanded numbers of kinases are a more general property of this phylum.

One striking exception to the overall similarity in the (normalized) distribution of kinases among the major subfamilies is the TKL kinase family (25) predicted for *Laccaria bicolor*. It is generally thought that there are no tyrosine kinases (TK) in fungi and our results support this. Nevertheless, in our data the Basidiomycete *Laccaria bicolor *shows, exceptionally, a huge number of TKL protein kinase genes. Pending direct experimental evidence, of course, this suggests a large deviation of this fungus kinome towards the TKL group.

### Kinome and Proteome Size

**Figure 2:**
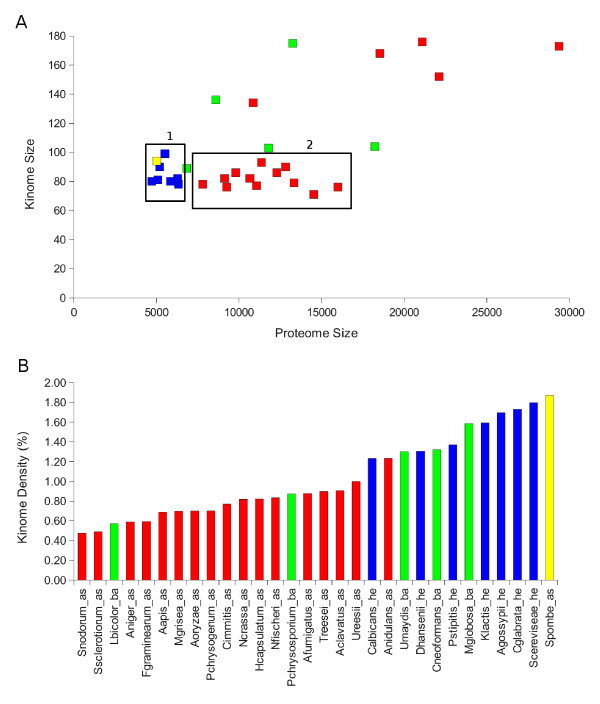
**Correlation between kinome size and proteome size**. Colors are as in Figure 1. A) Scatter plot: two clusters that can be inferred from this plot are indicated. B) Kinome density values (total number of kinases/proteome size).

An expanded and/or diverse kinome may provide a more flexible signaling network. This implies that overall parameters like kinome size as compared to proteome size might not always follow classical (and molecular) taxonomy. A striking example comes from recent work on the genomes of myxobacteria, which are prokaryotic, but contain an unexpectedly high number of eukaryotic-like (serine/threonine and tyrosine) protein kinases [15]. The proteome and kinome sizes of the fungi used in this study vary considerably. We note that the predicted fungal proteomes are only as accurate as the assumptions used in their construction. For example alternative splicing, a major source of protein diversity, is not taken into account, although there is evidence for biologically important alternative splicing events in fungi (in the *Neurospora *circadian clock, for example, [16,17]). Figure 2A shows the correlation between proteome and kinome sizes. The Hemiascomycota (Ascomycota, Saccharomycotina) fungi form a fairly uniform cluster (blue squares on cluster 1, Figure 2B). These species have smaller proteomes and tend to be highly similar in terms of kinome and proteome size. The Ascomycota (Pezizomycotina) and Basidiomycota groups have larger proteomes and also show greater variation; a subset of the filamentous Ascomycetes forms a compact group, quite variable in proteome size, but with very similar kinome sizes (cluster 2, Figure 2A).

Another way to look at this information would be to calculate the kinome density. This is the percentage of the total number of predicted kinase proteins within the total number of proteins predicted for each fungal species. Figure 2B shows the kinase density for all the fungi, sorted by value. It suggests that filamentous ascomycetes and the hemiascomycete yeasts can be classified according to their kinome density. The basidiomycetes have a much more variable kinome density and therefore it would be difficult to classify them based on this parameter. A Wilcoxon test (p-value = 9.6 × 10^-4^) confirmed that the Ascomycete and Hemiascomycete fungi could be successfully classified using their kinome density values. This difference in kinome density might represent the result of evolutionary pressure toward diversification of signal transduction pathways. Although this seems logical, there is really no obvious correlation between kinome density or diversity and the "lifestyle" (pathogen or saprophyte, particular host or ecological niche) of the sequenced species that we have studied. *S. pombe *is the only exception within the Ascomycetes, having the largest kinome density of all fungi studied. *S. pombe *though is the only species belonging to the Taphrinomycotina subphylum within the Ascomycetes, which represents its own subphylum branch. Thus we cannot anticipate whether this is a unique difference or a trend of its subphylum (see Figure 1 in [18]). Interestingly, the second densest fungal kinome belongs to *S. cereviseae*, another type of yeast.

### Domain distribution

Most protein kinases act in combination with other kinases and other signaling effectors, and are modulated by phosphorylation cascades. Other domains within these proteins have important regulatory activity, link to other signaling modules, or provide a localization signal [6]. We therefore studied the identity and number of domains flanking the kinase catalytic domain ePK in each predicted kinase. To this aim we searched the putative kinases against the PFAM database (see Methods).

**Figure 3:**
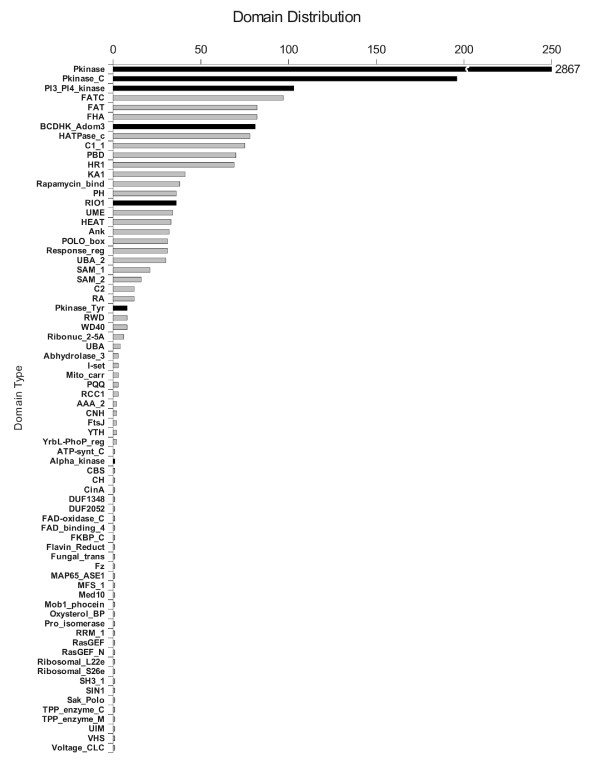
**Distribution of number of domains found in putative kinases over all fungal species**. Domains with kinase catalytic activity are colored black, others in grey.

The 30 fungal species have a total of 2976 putative kinase sequences, matching 4294 significant PFAM domains, which makes an average of 1.4 domains per kinase. According to PFAM, 3292 domains have kinase catalytic activity and the remaining 1002 have non-kinase domain activity. This suggests that the kinase proteins have an enormous richness of functional domains, with an average 0.3 of non-kinase domains and 1.1 kinase domains per sequence. We find that there are a total of 72 different domain types, of which 7 are annotated in PFAM to have kinase activity (see Figure 3 and text below): Pkinase (2867 domains), Pkinase_C (196), PI3_PI4_kinase (103), BCDHK_Adom3 (81), RIO1 (36), Pkinase_Tyr (8) and Alpha_kinase (1). The Pkinase domain is the most common type of kinase domain in our fungi, where it represents about 87% of all catalytic domains, and in PFAM, with more than 32000 representative sequences. But Pkinase is not the only conserved kinase catalytic domain type. There are 425 additional putative kinases with a catalytic kinase domain different from the classical kinase catalytic domain Pkinase: Pkinase_C is a kinase C terminal domain, PI3_PI4_kinase is a phosphatidylinositol 3- and 4-kinase domain, BCDHK_Adom3 is a mitochondrial dehydrogenase kinase domain, RIO1 is a typical serine kinase domain, the Pkinase_Tyr kinase, a tyrosine kinase domain and Alpha_kinase an alpha kinase domain. Those additional kinase domains represent almost 13% of the catalytic kinase domains, and add a rich variety of specific kinase catalytic functions to the kinome.

Very interestingly, while Pkinase (and in smaller proportion Pkinase_C, PI3_PI4_kinase, BCDHK_Adom3, HATPase_c and RIO1) appears almost in every fungal species, Pkinase_Tyr and Alpha_kinase domains are much more rare (see below). Pkinase_Tyr is highly represented only in *Laccaria bicolor *while isolated TK and TKL kinases were found in several species in our analysis and in the Kinomer database [10].

Figure 3 shows the sum of the domain distribution found in the predicted kinase proteins studied here. Overall, we have found that in addition to the 7 kinase domains, there are 65 different types of domains. This number is about three quarters of that found for the human kinome [6]. The three most common domains are kinase catalytic domains (in black in Figure 3), while many of the highly frequent non-kinase domains (in grey in Figure 3) are kinase regulatory domains, like FHA, FATC, HR1, etc. Additionally, Figure 3 shows that there are 32 very rare domains, appearing only once in all the kinomes studied, like TPP_enzyme_C (Thiamine pyrophosphate enzyme, C-terminal TPP binding domain) and Fungal_trans domain (Fungal specific transcription factor domain). This variation in the domain type and frequency indicates functional and evolutionary differences that are not easy to interpret, but that can, in principle, be used to classify the fungi, as a complement to classical taxonomical and phylogenetic procedures.

Of the first 10 most common domains in the fungi studied here, 9 are also present in the human kinome, reflecting a highly conserved kinome functional milieu: Pkinase_C (Catalytic kinase), PI3_PI4_kinase (Catalytic kinase), FATC and FAT (Accessory domain for PI34K domains), FHA (Nuclear signaling), HATPase_c (ATPase catalytic activity), C1_1 (Phospholipid binding) and PBD (GTPase interaction). These functional domains have very broad and general functions, thus explaining why they appear in all types of fungi and also in the human kinome. The exception is BCDHK_Adom3; this domain is involved in the regulation of the dehydrogenase complex that breaks down branched-chain amino-acids and it is similar to the HATPase_c family [19]. Interestingly, however, these are, as mentioned, the 10 most common accessory domains in fungi, while in human only two of them, Pkinase_C and C1_1, are also among the 10 most common domains. The remaining six domains that do exist in the human kinome are far less common than in fungi, indicating that these kinase associated domains could be good indicator of functional differences among species.

We believe that the kinome domain distribution, both in type and number, should be indicative of functional and evolutionary differences between fungi. In the next section, we show that this variability is enough to differentiate among different fungal phyla and subphyla.

### Domain analysis and Principal Component Analysis grouping

**Figure 4:**
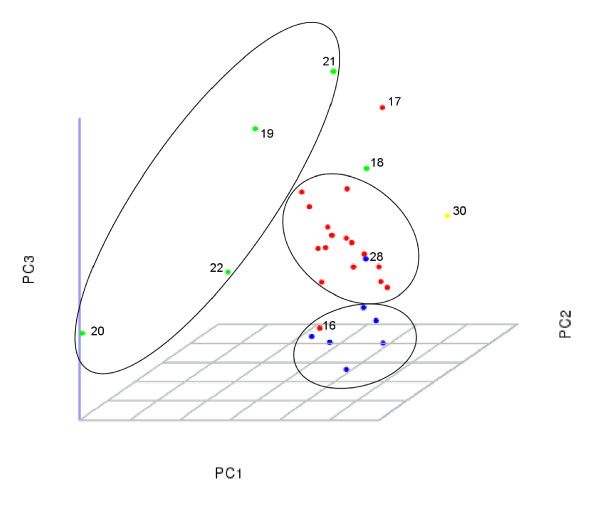
**Principal Component Analysis (PCA) of domain frequencies**. The PCA plot is based on 21 domain frequencies in all 30 fungi (*i.e*. domains present in at least half of the fungi, see Additional File 4), projected onto three uncorrelated axes (principal components). The PCA analysis revealed two tight clusters for Ascomycota/Pezizomycota (red dots) and Saccharomycotina (blue dots) groups, and a more spread, but still distinguishable, cluster for Basidiomycetes (green dots). Ellipsoids enclose the clusters for clarity. The only representative of Ascomycota/Taphrinomycotina (*S. pombe*) dot 30 (in yellow), forms its own singleton cluster. Species numbers (according to Table 1) are given for some dots, for reference in the text.

Taxonomic classification is not always a straightforward task. In this work we tested several criteria for clustering all 30 fungi studied here, based on the number and type of kinases and additional domains (see Methods). Following that, we compared the results with fungal taxonomy [20,21]. We found that the most informative clustering was achieved when considering only the distribution of the most common accessory domains. Figure 4 shows the PCA clustering of the different fungi, based on the frequency and type of the 21 most common domains found among all fungal kinomes (see Methods). This classification yields 3 clusters of fungi which show a high correspondence with classical taxonomic classification.

As illustrated in Figure 4, the PCA grouping shows that the domain distribution is more similar among the Pezizomycota subphylum of the Ascomycota and among the Saccharomycota subphylum of the Ascomycota. Among the Basidiomycetes the domain distribution is less tight, although still distinguishable from the first two groups. Though only five species are currently available for analysis, it is noticeable that *M. globosa *and *U. maydis *both belong to the subphylum Ustilaginomycotina and are fairly close to each other (dots 20 and 22). Likewise, dots 18, 19 and 21, which represent members of the Agaricomycotina: *C. neoformans*, *P. chrysosporium *and *Laccaria bicolor*, are closely located. Assuming that *S. pombe *forms its own singleton cluster, 26 members of the fungi family in this study are clustered by the PCA into one of four groups according to their domain distribution. Four species fall outside these clusters. Of those, two do not reside in any group, and two are mixed: among the ascomycetes, one member of the Pezizomycota (dot 16, Figure 4) and one member of the Saccharomycotina (dot 28, Figure 4) crossed to the other side of the cluster boundaries. Very interestingly, here again *S. pombe *(Ascomycota/Taphrinomycotina), clusters differently from the Pezizomycotina and Saccharomycotina groups. Indeed, when looking closely at the domain content, there are several examples in which *S. pombe *is quite different from its ascomycete relatives. For example, it has a much lower density of HATPase_c domains, a higher percentage of HEAT domains and a higher percentage of the Rapamycin_bind domains. It would be difficult to conclude whether this reflects any aspect of fission yeast lifestyle, or is simply a consequence of evolutionary distance among the species. The PCA results indicate clearly that the domain distribution includes different evolutionary information at the level of the subphyla.

While the classification, based on the most common functional domains from the kinome, is very similar to the classical taxonomy it would also be interesting to try and understand the functional and evolutionary implications of the rare domains appearing only in a certain phyla or fungal species. For example, the ascomycete *A. nidulans *has unique domains like CNH, FAD-oxidase_C, RCC1 and many more, making a total of 16 unique accessory domains, which suggests that *A. nidulans *has possibly acquired a diverse kinase-related functionality. Currently, based on its physiology, there is no obvious clue as to why this should be so, but since *A. nidulans *is one of the best-studied model genetic species there may be a good basis to understand this result in future studies.

From the most common domain distribution, there are some clear cases of variations between the subgroups. For example the POLO_box domain appears in all Basidiomycota and in the Saccharomycotina, while is quite rare within the filamentous Ascomycota. Polo boxes appear to mediate interaction with multiple proteins through protein-protein interactions. The HEAT domain, common in both Ascomycete subphyla Pezizomycotina and Saccharomycotina, is extremely rare in Basidiomycetes (appears only in *C. neoformans*). Many HEAT repeat-containing proteins are involved in intracellular transport processes. Although we cannot fully understand how these differences directly impact the function of the different species, there is a clear correlation between the domain distributions and the taxonomic classification.

## Conclusions

The overall distribution of protein kinases within very different fungal phyla and subphyla seems to be very similar. The overall kinome density is in good agreement with taxonomy. The distribution of additional domains, which could have functional implications, does differ significantly between species, and seems able to provide a functional classification that overlaps with taxonomical classification. Although generally the classical phyla classification correlates with the kinome density and domain distribution, there are exceptions. Basidiomycota do not cluster by kinase number, but they have a similar kinome to proteome ratio. Ascomycetes are well clustered by all criteria, with two exceptions: *A. nidulans *has a different kinome to proteome ratio and a different kinase distribution. Nevertheless, *A. nidulans *is not unusual according to the PCA analysis. Among the filamentous ascomycetes, there is no obvious clustering according to class within the subphylum Pezizomycotina. We note, however, that the class Eurotiomycetes is over-represented in the sequenced genomes published to date, perhaps because the beneficial (*Penicillium, Aspergillus oryzae*), harmful (*Aspergillus nidulans*), or pathogenic (*Coccidioides*, *Histoplasma*) members of this group, which have drawn much attention over the years. *Schizosaccharomyces pombe *has a very high kinome density much similar to the Saccharomycotina group. The predicted proteome of *Laccaria bicolor *has an extraordinary number of TKL kinases; further work can determine whether this is an anomaly, or a more general trait found in mycorrhizal symbionts [22]. Finally, the PCA approach based on the most common domains clusters the Pezizomycotina group and the Saccaromycotina group very tightly, while the Basidiomycetes are more divergent. The approach taken here could be repeated for additional groups of proteins (*e.g*. G-protein coupled receptors) in order to study their evolution and variability within each fungi phylum. These data can also be used to guide experimental work to elucidate the function of individual protein kinases and the signal transduction pathways they function in.

## Methods

### Kinase collection and analysis pipeline

**Figure 5:**
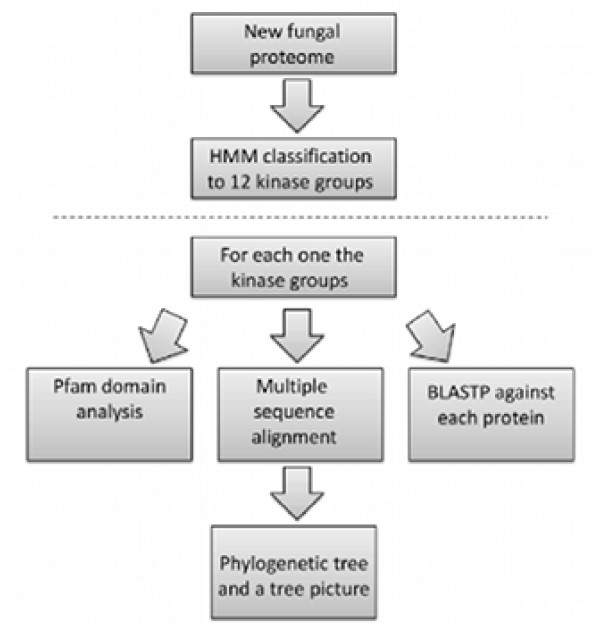
**Flow chart of the kinome analysis pipeline**.

We have designed and implemented an automatic pipeline (Figure 5) to extract all putative kinases from fungi proteomes and explore their properties. The pipeline uses a variety of tools to extract and classify the putative kinases from all the fungi. Below we describe the pipeline, which can be downloaded as Additional File 2.

### Kinase searching and classification

In order to extract the kinome we fed the HMMer software (version 2.3.2 [23]) with pre-calculated multilevel HMM libraries from the Kinomer 1.0 database. HMMer uses profile hidden Markov models (HMMs) to do sensitive database searching using statistical descriptions of a sequence family's consensus. This process allows an accurate extraction and classification of protein kinases into one of the 12 previously defined kinase groups [8]. Our procedure is very similar to that described by [8], the only significant difference is the cutoff value used for each putative kinase. While Miranda-Saavedra and coworkers [8] used a variable e-value cutoff to choose the correct group for each kinase, we used a fixed HMM bit score cutoff: if the score is greater than 20 a protein is predicted to be a kinase, and if more than one HMM is matched, the higher is selected. Miranda-Saavedra and coworkers reported a high accuracy of their HMM, which according to their study are able to identify successfully between 90 and 97.5% of all the ePKs of 37 annotated kinomes; from those ~98% were estimated to be correctly classified in each of the subgroups [8]. Therefore we believe that our results should present a similar accuracy, despite the fact that there might be a few mis-classifications in any group of kinases. The modified cutoff criteria used here (see Methods) resulted in slightly different numbers of predicted kinases when compared to the Kinomer results, but the calculated distribution (data not shown) is very similar, for those species previously studied [8].

We chose a fixed bit score cutoff, since it provides us with a unified criterion for all fungi, so we can compare the numbers based on the same scale. The bit score reflects whether the sequence is a good match to the HMM model. A score above log_2 _of the number of sequences in the target database is likely to be a true homologue. For our fungi proteomes, this rule-of-thumb number is on the order of 20 bits. In any case e-value and bit score should be strongly correlated, true homologues will have both a good bit score and a good E-value [23].

Once the kinase groups are populated, the pipeline is designed to extract functional and phylogenetic information from the list of putative kinases. The pipeline procedure and subsequent analysis (see below) is summarized in Figure 5. Since we preferred here to miss a number of potential kinases rather than including false positives, we did not use the category "Others" as described in the Kinomer database.

### Functional information

Homology search - The pipeline runs Blastp [24] (version 2.2.17) against the Swissprot database (November 2008 version) to allow comparison to known proteins with annotation. The output of this stage is a list of all homologs for each kinase entry. Data from this stage is not shown in the paper.

Domains search - The pipeline identifies the domains of each putative kinase by running the pfam_search.pl script against PFAM A [25] HMMs library. We then analyze the presence of each domain within a specific fungal group.

### Phylogenetic information

In order to build phylogenetic trees we constructed multiple alignments for each group using the MSA program MUSCLE (version 3.7) [26,27]. The multiple sequence alignments are then used for constructing phylogenetic trees using FastTree version 2.0 [28] with the generalized time-reversible models of nucleotide evolution and the JTT model [29] of amino acid evolution. We used FigTree http://tree.bio.ed.ac.uk/software/figtree/ for phylogenetic visualization. Additional File 3 shows one example of the phylogenetic trees produced. This corresponds to the 134 predicted kinases of *Aspergillus nidulans*. Aside from a few exceptions, the kinases are clustered according to their predicted group.

The pipeline is written in Perl scripting language and was tested on Fedora and Ubuntu operating systems.

### Principal Component Analysis (PCA)

We classified the 30 fungi based on the percentage of each domain type found in each species, limiting the data to those domains present in at least half of the fungi (see Additional File 4). We then applied the PCA procedure described in [30] to cluster the fungi. The PCA was obtained using the GNU R software (R: A Language and Environment for Statistical Computing, http://www.R-project.org, 2009).

### Significance test

The Wilcoxon-Mann-Whitney test was performed on the kinome density values of our three phylogenic groups using GNU R software (R: A Language and Environment for Statistical Computing, http://www.r-project.org/index.html) and the wilcox.test function with its non paired mode.

## Authors' contributions

IK participated in the design of the study, set up and tested the computational tools and did the major part of the analysis; YMG participated in the design and coordination of the project and in data interpretation; BAH initiated our interest in fungal protein kinases and provided the biological context; FG conceived of the bioinformatics study, designed it, and did the domain analysis. FG and BAH drafted the manuscript; IK and YMG helped draft the manuscript. All authors read and approved the final manuscript.

## Authors' information

IK is a graduate student in the structural biology and bioinformatics lab led by YMG at the Department of Biology, Technion - Israel Institute of Technology. BAH, also at the Department of Biology, Technion, leads a lab studying signal transduction pathways of filamentous fungi, and is currently involved in annotation for several fungal genome projects. FG is a staff member of the Bioinformatics Knowledge Unit at the Technion - Israel Institute of Technology.

## Supplementary Material

Additional File 1 **Total predicted number of kinases per fungal species**. Bar colors indicate significantly different kinome size (see text for details). Click here for file

Additional File 2 **Pipeline for kinome analysis**. This file contains the pipeline software and documentation. Click here for file

Additional File 3 **Phylogenetic tree of the *Aspergillus nidulans *kinome**. Taxa names are composed of the predicted kinase group followed by an underscore and the protein code as it appears in the original proteome. Simulated bootstrapped values are also shown. Click here for file

Additional File 4 **Domain percentage data used as input for the PCA analysis**. The first column lists the species names, abbreviated according to Table 1. The values are the percentages of the domains indicated in each column, according to PFAM notation. Click here for file
