# Supplementary figures and images for: Comparative analysis of fungal protein kinases and associated domains
Source: BMC Genomics. 2010 Feb 24;11:133. doi: 10.1186/1471-2164-11-133 (PMC2838846; doi:10.1186/1471-2164-11-133)

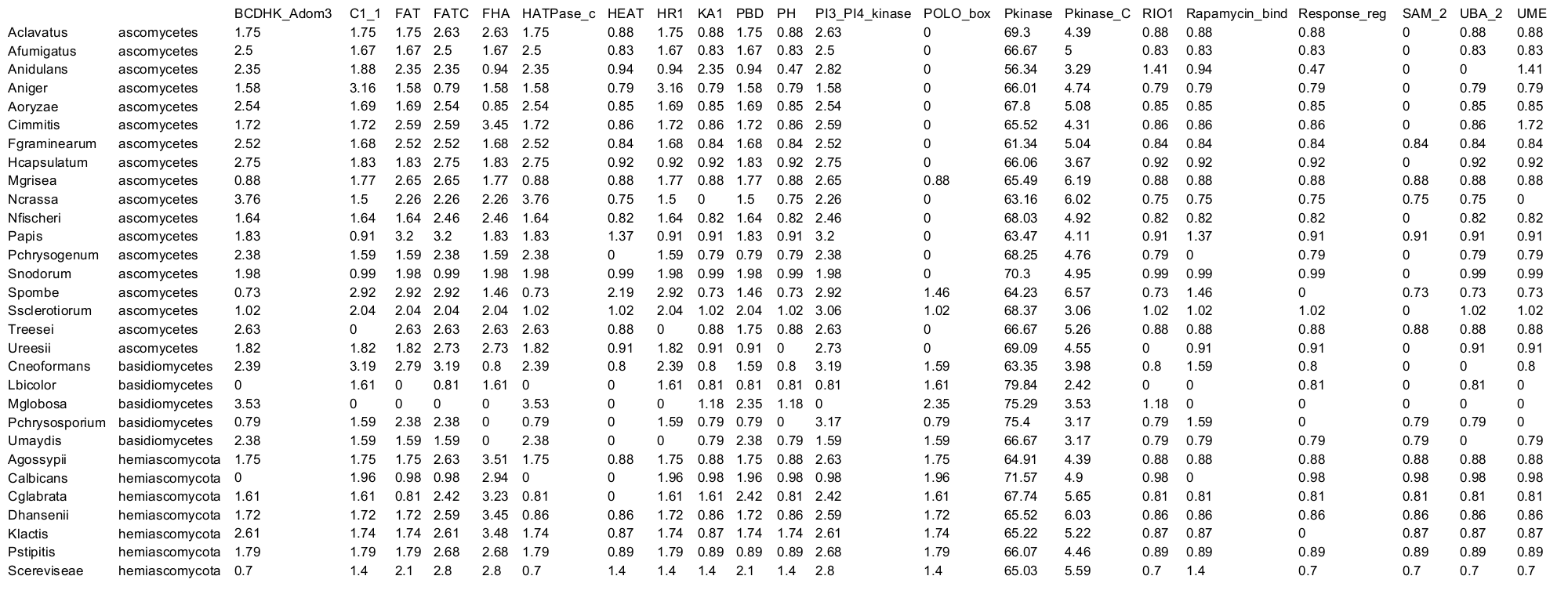

Supplement: Additional File 4 — Domain percentage data used as input for the PCA analysis. The first column lists the species names, abbreviated according to Table 1. The values are the percentages of the domains indicated in each column, according to PFAM notation. [file 1471-2164-11-133-S4.PNG]
